# Comparing the trends of cancer burden attributed to high BMI in China and globally from 1990 to 2021, with multi-model prediction to 2036

**DOI:** 10.3389/fpubh.2025.1590559

**Published:** 2025-07-11

**Authors:** Nanting Chen, Lingzhi Xing, Fengyun Xiang, Chengmiao Li, Letai Li, Jingsong Cheng, Yangfan Yu, Yubowen Gong, Xiao Liu, Fangjiao Xie, Ling Chen

**Affiliations:** ^1^The Second Clinical College, Chongqing Medical University, Chongqing, China; ^2^The Center of Experimental Teaching Management, Chongqing Medical University, Chongqing, China; ^3^Faculty of Pediatrics, Chongqing Medical University, Chongqing, China; ^4^The Fifth Clinical College, Chongqing Medical University, Chongqing, China; ^5^The First Clinical College, Chongqing Medical University, Chongqing, China; ^6^Key Laboratory, Tongliang District People's Hospital, Chongqing, China; ^7^The Department of Clinical Laboratory, The People's Hospital of Rongchang District, Chongqing, China

**Keywords:** global burden of disease, high BMI, cancers, age-standardized mortality rate, age-standardized disability-adjusted life-years rate, China

## Abstract

**Introduction:**

High body mass index (BMI) has been identified as a significant contributor to cancers. However, details regarding the evolution of the cancer burden attributable to high BMI in China have not been available. With the epidemic of high BMI among Chinese recent years, it's essential to evaluate the disease burden of cancer associated with high BMI to guide disease interventions and enhance public health. This study aimed to evaluate the burden of high BMI-attributed cancer in China from 1990 to 2021 and compare it with global trends.

**Methods:**

The temporal trends of high BMI-attributed cancer were assessed using annual percentage change (APC) and overall percentage change. Decomposition and age-period-cohort analyses were conducted to identify influential factors, while future trends were projected with the Bayesian age-period-cohort (BAPC), auto-regressive moving average model (ARIMA), and exponential smoothing model (ETS).

**Results:**

In China, the age-standardized mortality rate (ASMR) and age-standardized disability-adjusted life-years rate (ASDR) for high BMI-attributed cancer increased to 2.81 (95% UI: 1.20–4.76)/10^5^ and 79.17 (95% UI: 33.82–134.14)/10^5^ in 2021, remaining below the global average. While the APC of ASMR and ASDR constantly increased in China, global trends exhibited minimal change. Colorectal and liver cancers were the most prevalent types of high BMI-attributed cancer. In China, the period and cohort effects on high BMI-attributed cancer increased more significantly, with the age effect showing an exponential rise. Aging accounted for 43.92% of high BMI-attributed cancer related deaths and 40.03% of disability-adjusted life-years (DALYs) in China. Over the next 15 years, the burden of high BMI-attributed cancer in China would show a more significant upward trend compared with global trends.

**Conclusions:**

Although China's current high BMI-attributed cancer burden remains below the global average, it is increasing at a substantial rate and is expected to continue increasing rapidly. Targeted prevention strategies tailored to age and the latest high BMI-attributed cancer spectrum are urgently needed to mitigate this growing public health concern in China.

## 1 Introduction

The global burden of cancer is escalating at an alarming rate. Projections from GLOBOCAN 2020 indicate an anticipated 47% increase in cancer cases from 2020 to 2040 (1). In 2020, global cancer-related deaths reached nearly 10.0 million, with China accounting for 30% (3.0 million) of these deaths (2), and China ranked first in cancer incidence and mortality in the world (3).

Primary prevention is crucial in alleviating the cancer burden. High body mass index (BMI) is a significant risk factor for various types of cancer and is a main modifiable cause of cancer (4–7). An American study found that overweight and obesity contributed to 10.9% of new cancer cases in women and 4.8% in men (8). Prior research on high BMI-attributed cancer has mainly concentrated on high-income nations, including the USA and various European countries (9–12). Information on the changes in cancer burden attributed to high BMI in China has been lacking. In the last 10 years, Chinese adults have experienced a significant rise in BMI and obesity rates. Between 2004 and 2018, the average BMI of Chinese adults increased from 22.7 kg/m^2^ to 24.4 kg/m^2^, with the obesity rate rising from 3.1% to 8.1%. From 2010 to 2018, the average annual BMI growth was 0.09 kg/m^2^ (13, 14). Assessing the cancer burden attributed to high BMI is essential for guiding targeted interventions and improving public health strategies in China.

We analyzed trends and influential factors in the high BMI-attributed cancer burden in China and globally from 1990 to 2036 using the recent Global Burden of Disease (GBD) database. Global trends in high BMI-attributed cancer burden serve as a benchmark for assessing China's temporal trends and the key influencing factors of high BMI-attributed cancer. Three time series models were used to project the future burden of high BMI-attributed cancer until 2036. This analysis aims to provide a foundation for disease prevention and the accurate development of policies in China and similar countries.

## 2 Methods

### 2.1 Data sources

We employed repeated cross-sectional data from the Global Health Data Exchange for our analysis of GBD2021. We used “GBD Results” tool updated on May 16, 2024 to obtain our data (https://vizhub.healthdata.org/gbd-results/, access period: 2024-08-26 to 2024-10-05). The study population comprised individuals diagnosed with various types of cancer in China and globally. We extracted data about disability-adjusted life-years (DALYs), age-standardized mortality rate (ASMR), age-standardized disability-adjusted life-years rate (ASDR) and summary exposure value (SEV) of high BMI-attributed cancer among adults (≥20 years) from GBD2021. SDI (sociodemographic index) and population data of different nations and regions from 1990 to 2021 were also derived from GBD2021. The predicted population was obtained from the World Population Prospects 2022 (15).

### 2.2 Definitions

The GBD2021 study assessed the impact by contrasting actual health outcomes with hypothetical scenarios based on historical exposure. A high BMI for individuals aged 20 and older is classified as exceeding 25 kg/m^2^ (16). High BMI attributed cancer referred to 12 kinds of cancers whose occurrence and development are associated with elevated BMI levels. The international classification of disease codes for these cancers in GBD2021 is available in related publications and on the online platform (17).

The GBD study assessed risk factor prevalence using SEV, adjusted by relative risk. In terms of relative risk, a value of zero indicates no excess risk for the population, whereas a value of one represented the highest level of risk. In the GBD study, DALYs served as comprehensive indicators for assessing the disease burden from disability and early mortality (16). DALYs are determined by adding the years of life lost to the years lived with disability. SDI is considered a comprehensive indicator of development, strongly correlated with health outcomes. In GBD2021, the final SDI values were scaled by a factor of 100 for reporting purposes. An SDI of 0 indicates the lowest theoretical level of health-related development, whereas an SDI of 100 denotes the highest theoretical level.

### 2.3 Risk-attributable burden

The GBD2021 utilized a comparative risk assessment framework to evaluate the link between high BMI and cancer. Since 2002, the GBD has employed this method to systematically conduct meta-analyses of the data. This method could eliminate the influence of confounding factors to a certain extent (18, 19). Exposure data for each risk factor were modeled using Spatiotemporal Gaussian process regression or DisMod-MR 2.1 (20). Both are established Bayesian models commonly used in GBD analyses.

### 2.4 Statistical analysis

Joinpoint analysis, as proposed by Kim et al. (21), was used to evaluate the ASMR and ASDR of high BMI-attributed cancer from 1990 to 2021. The model can avoid the non-objectivity defect that occurs in linear trends. The annual percentage change (APC) and its 95% confidence interval (CI) were calculated for each segment to assess the magnitude and direction of trends in ASMR and ASDR. An increasing trend in ASMR and ASDR is indicated when the lower limit of the 95% CI for the APC exceeds zero, and a decreasing trend is indicated when it is below zero. Joinpoint regression was conducted utilizing Joinpoint software (Version 5.4.0).

Decomposition analysis identifies factors contributing to changes in the absolute number of age-related disease burdens (22–24). We performed a decomposition analysis to evaluate changes in mortality and DALYs from 1990 to 2021, considering age structure, epidemiological shifts, and population size.

An age-period-cohort model analysis was performed to assess the impacts of age, period and cohort on high BMI-attributed cancer. The age effect encompasses alterations in physiological, pathological, and social conditions due to aging, while the period effect represents variations in the burden of high BMI-attributed cancer, shaped by factors like advancements in diagnosis, treatments, and healthcare reforms. The cohort effect pertains to differences in lifestyle and exposure to risk factors among different generations. The age, period, and cohort effect coefficients were estimated using the age-period-cohort model with the intrinsic estimator method (25). This study utilized the age-period-cohort analysis tool available at https://analysistools.cancer.gov/apc/ (26). Statistical significance was determined by *P-values* < 0.05 (two-tailed).

To project the ASMR and ASDR of the high BMI-attributed cancer burden from 2022 to 2036, we applied three methods: Bayesian age-period-cohort (BAPC), auto-regressive moving average (ARIMA), and exponential smoothing (ETS) model. Prior research shows that BAPC excels in predicting non-communicable diseases, especially for short-term forecasts (27, 28). The ARIMA and ETS models are also commonly used models for predicting the epidemiological situation of cancers (29–31). We adopted the BAPC model with integrated nested Laplace approximations to analyze historical data and estimate high BMI-attributed cancer burden. ARIMA combines autoregressive and moving average models. It assumes data series are time-dependent random variables whose autocorrelation can predict future values based on past values (32). The ETS model forecasts by weighted averaging historical data, assigning higher weights to recent data. Exponential smoothing fits models by combining error, trend, and seasonality components via addition, multiplication, or no operation (33). We split the dataset into a 70% training set and 30% test set. After fitting the three models on the training data, we tested them on the test set. Using mean absolute error (MAE), mean absolute percentage error (MAPE), and root mean square error (RMSE) as performance metrics, we aimed to identify the optimal model (34).

## 3 Results

### 3.1 Analysis of temporal trends in cancer burden attributed to high BMI in China and globally

**Figure 1:**
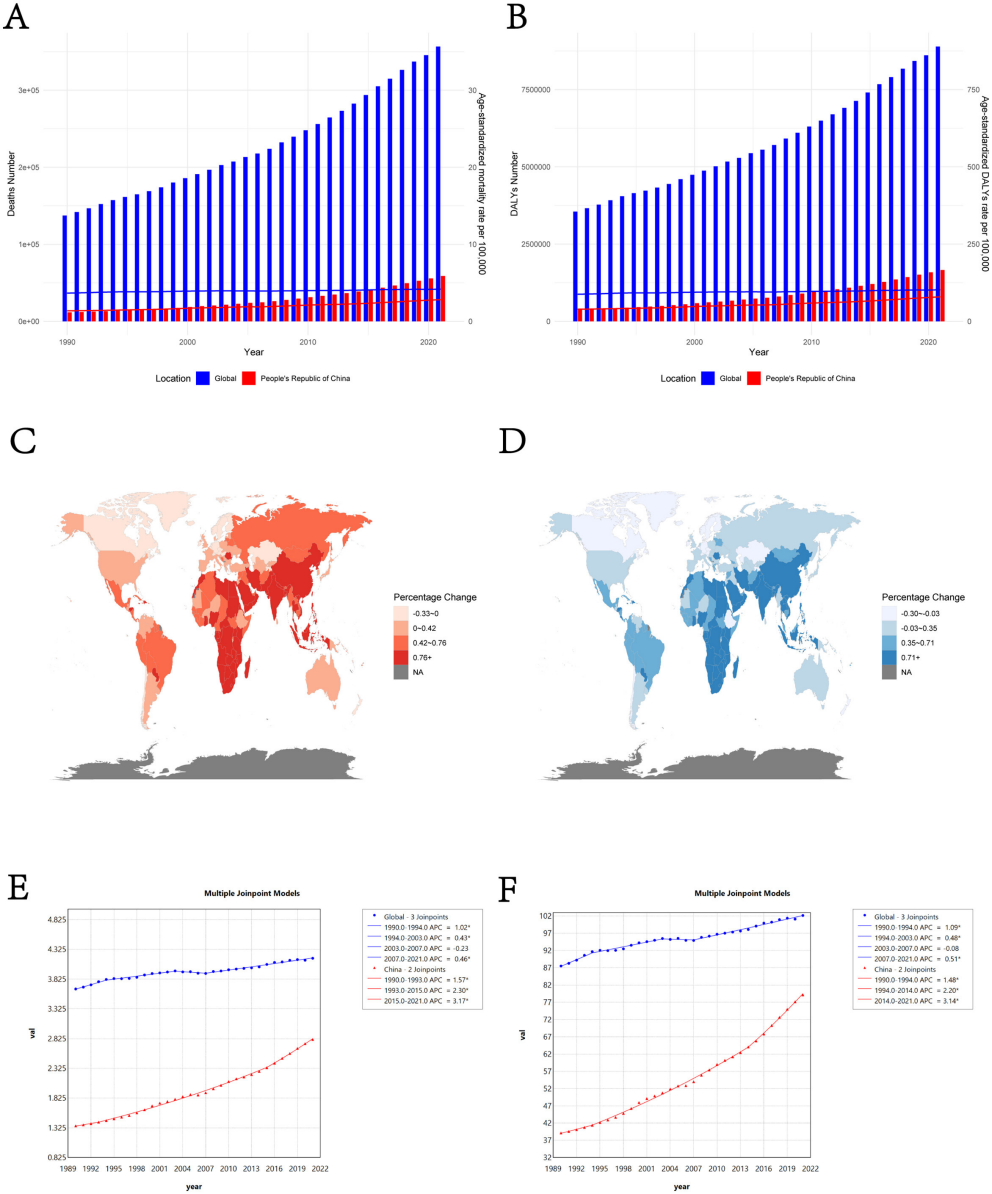
**(A)** Trends in high BMI-attributed cancer death number and ASMR in China and worldwide from 1990 to 2021; **(B)** Trends in high BMI-attributed cancer's DALYs and ASDR in China and globally from 1990 to 2021. The bar graphs depict the quantity, whereas the line graphs illustrate the age-standardized rates. The percentage change in ASMR **(C)** and ASDR **(D)** due to high BMI-attributed cancer across 204 countries and territories from 1990 to 2021. Joinpoint regression analysis of ASMR **(E)** and ASDR **(F)** for high BMI-attributed cancer in China and worldwide from 1990 to 2021.

Figure 1 illustrates the trends in deaths and DALYs of high BMI-attributed cancer in China and globally from 1990 to 2021. The findings indicate that China's ASMR and ASDR for high BMI-attributed cancer remain below the global average (Figures 1A, B). SDI is strongly associated with diseases caused by high BMI in previous studies (35). Consistent with this, our study revealed that areas with higher SDI showed a decline in ASMR, whereas areas with lower SDI showed an increase (Supplementary Figures S1A, B). Furthermore, a comparison of China's high BMI-attributed cancer burden with countries of similar SDI levels revealed that China's ASMR and ASDR were significantly lower than anticipated based on SDI alone (Supplementary Figures S1C, D).

From 1990 to 2021, the temporal trend analysis revealed that the burden of high BMI-attributed cancer exhibited an overall increasing trend in China and globally. Table 1 presents the changes in mortality numbers, ASMR, DALYs, ASDR, and SEV in China and globally between 1990 and 2021. In 2021, an estimated 58,745 (95% UI: 24601–99889) people in China died from high BMI-attributed cancer, representing nearly five times compared with 1990. The ASMR increased from 1.36 (95% UI: 0.69–2.15)/10^5^ in 1990 to 2.81 (95% UI: 1.20–4.76)/10^5^ in 2021, and ASDR increased from 39.14 (95% UI: 20.46–61.54)/10^5^ in 1990 to 79.17 (95% UI: 33.82–134.14)/10^5^ in 2021. Globally, high BMI-attributed cancer related deaths in 2021 totaled 356,738 (95% UI 146116–581012), marking a 159.72% rise since 1990. The number of DALYs of high BMI-attributed cancer globally increased by 150.62% compared to 1990. The ASMR increased from 2.58 (95% UI: 1.08–4.22)/10^5^ in 1990 to 4.52 (95% UI: 1.85–7.36)/10^5^ in 2021, and ASDR increased from 87.53 (95% UI: 37.43–141.83)/10^5^ in 1990 to 102.17 (43.24–165.02)/10^5^ in 2021 (Figures 1 A, B; Table 1). Figures 1C, D illustrate the percentage change in ASMR and ASDR for high BMI-attributed cancer across 204 countries and territories from 1990 to 2021. Compared with other countries and territories, China was observed to have a more significant percentage change in terms of ASMR (1.07, 95% UI: 0.54–1.62) and ASDR (1.02, 95% UI: 0.49–1.60). The SEV of high BMI in China also increased rapidly from 1990 to 2021, which ranked as 6th among 204 countries and territories in terms of percentage change.

**Table 1:** Changes in H BAC mortality, ASMR, DALYs, ASDR (per 100,000), and SEV in China and globally form 1990 to 2021.

**Metric**	**Measure**	**China**			**Global**		
		**1990 *n* (95%UI)**	**2021 *n* (95%UI)**	**Percentage Change *n* (95%UI)**	**1990 *n* (95%UI)**	**2021 *n* (95%UI)**	**Percentage change *n* (95%UI)**
SEV	All ages (%)	7.53 (6. 37–9. 33)	20.13 (17.59–2399)	1.67 (1.3–1. 97)	11.87 (10.59–13. 84)	21.85 (19.44–24.49)	0.84 (0.72–0.92)
	Age-standardized (%)	8.75 (6.58–9.65)	19.09 (17.11–22.15)	1.46 (1.13–1. 7)	12.58 (11.14–14.74)	21.49 (19.22–23.98)	0.71 (0.61–0.79)
Death	Numbers (n)	11556 (6040–18285)	58745 (24601–99889)	4.08 (2.69–5.54)	137353 (57450–225297)	356738 (146116–581012)	1.6 (1.42–1.73)
	Mortality (All ages,1/10^5^)	0.98 (0.51–1.55)	4.13 (1.73–7.02)	3.2 (2.05–4.41)	2.58 (1.08–4. 22)	4.52 (1.85–7. 36)	0.76 (0.63–0.85)
	ASMR	1.36 (0.69–2.15)	2.81 (1.2–4.76)	1.07 (0.54–1.62)	2.66 (1. 51–6. 03)	4.18 (1.71–6. 8)	0.14 (0.07–0. 2)
DALYs	Numbers (*n*)	377746 (203505–588229)	1658721 (693186–2831308)	3.39 (2.13–4.68)	3549049 (1548429–5731481)	8894525 (3751953–14385271)	1.51 (1.29–1.64)
	All-age rate (1/10^5^)	32.11 (17.3–50)	116.59 (48.72–199)	2.63 (1.59–3.69)	66.54 (29.03–107.46)	112.71 (47.55–182.29)	0.69 (0.55–0.79)
	ASDR (1/10^5^)	39.14 (20.46–61. 54)	79.17 (33.82–134.14)	1.0 (0.49–1.6)	87.53 (37.43–141.83)	102.17 (43.24–165.02)	0.17 (0.08–0.23)

UI, uncertainty interval; SEV, summary exposure value; DALYs, disability-adjusted life-years; ASMR, age-standardized mortality rate; ASDR, age-standardized disability-adjusted life-years rate.

Joinpoint regression analyses were employed to further explore the changes speed of ASMR and ASDR in different time segments (Figures 1E, F; Supplementary Tables S1, S2). In China, the APC of ASMR and ASDR constantly increased in three segments (ASMR: APC_1990 − 1993_ = 1.57%, 95% CI: 0.01%−3.16%, APC_1993 − 2015_ = 2.30%, 95% CI: 2.21%−2.38%, APC_2015 − 2021_ = 3.17%, 95% CI: 2.57%−3.77%; ASDR: APC_1990 − 1994_ = 1.48%, 95% CI: 0.68%−2.28%, APC_1994 − 2014_ = 2.20%, 95% CI: 2.12%−2.28%, APC_2014 − 2021_ = 3.14%, 95% CI: 2.75%−3.54%). However, the APC of ASMR and ASDR globally changed relatively small across different segments and even showed negative values (ASMR: APC_1990 − 1994_ = 1.02%, 95% CI: 0.81%−1.38%, APC_1994 − 2003_ = 0.43%, 95% CI: 0.35%−0.52%, APC_2003 − 2007_ = −0.23%, 95% CI: −0.50% to 0.01%, APC_2007 − 2021_ = 0.46%, 95% CI: 0.42%−0.50%; ASDR: APC_1990 − 1994_ = 1.09%, 95% CI: 0.86%−1.51%, APC_1994 − 2003_ = 0.48%, 95% CI: 0.39%−0.58%, APC_2003 − 2007_ = −0.08%, 95% CI: −0.36% to 0.18, APC_2007 − 2021_ = 0.51%, 95% CI: 0.47%−0.57%).

### 3.2 The changes of high BMI-attributed cancer spectrum in China and globally

**Figure 2:**
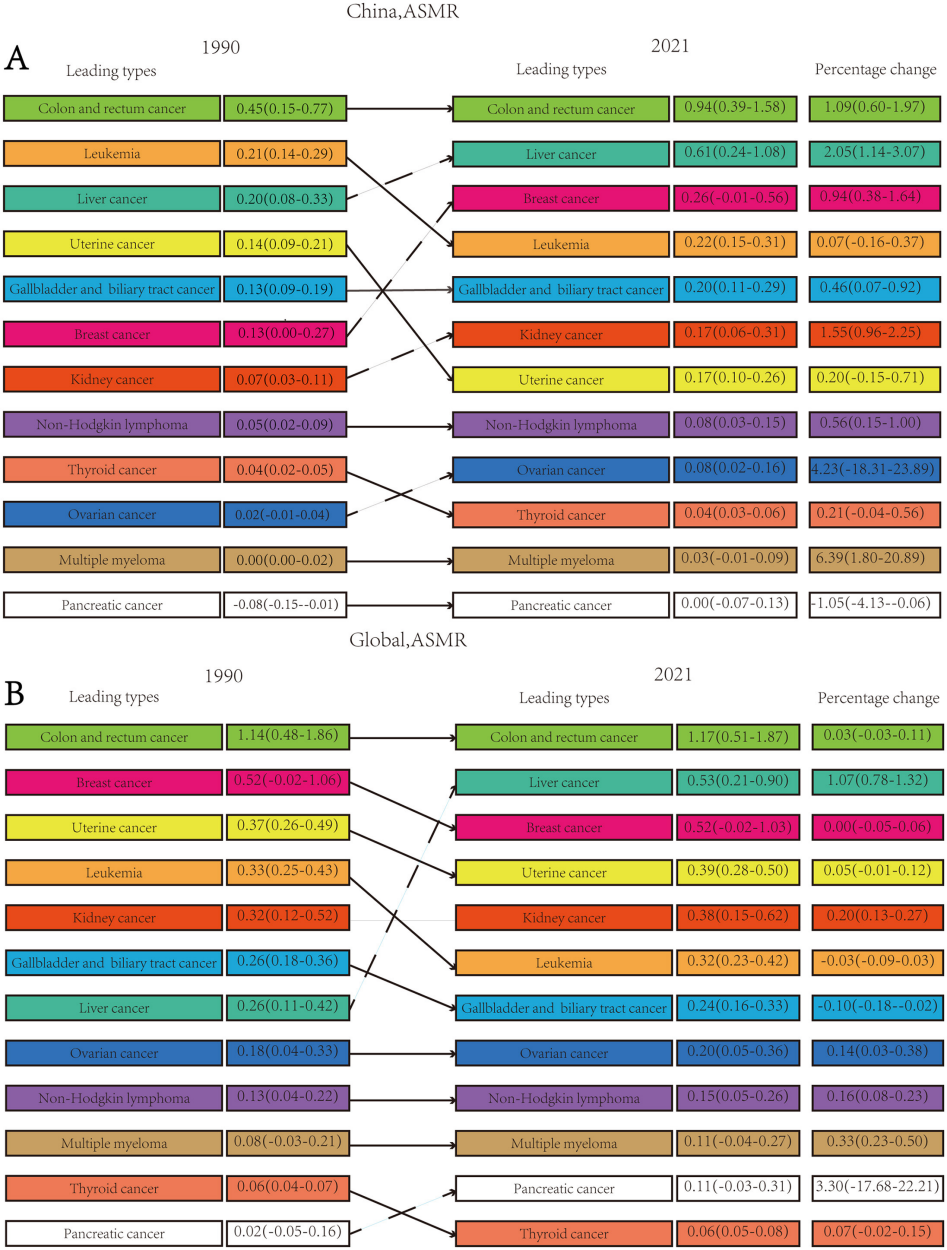
Comprehensive ranking of cancer categories attributed to high BMI in China and worldwide from 1990 to 2021. **(A)** ASMR ranking in China; **(B)** ASMR ranking in global. Solid lines represent either a decrease or no change in rank. Data in parentheses indicate the 95% UI.

From 1990 to 2021, the spectrum of high BMI-attributed cancer changed dramatically. Trends in ASMR and ASDR for different cancer types attributed to high BMI are presented in Figure 2 and Supplementary Figure S2. In 2021, the five leading obesity-related cancers in China, based on ASMR, were colorectal cancer, liver cancer, breast cancer, leukemia, and gallbladder and biliary tract cancer. Globally, the top five cancers with the highest ASMR were colorectal cancer, liver cancer, breast cancer, uterine cancer, and kidney cancer. Colorectal cancer attributed to high BMI represented the predominant contributor to the total high BMI-attributed cancer burden in both 1990 and 2021. In China, it accounted for 33.1% (19,418) deaths and 30.6% (507,316) DALYs of high BMI-attributed cancer. Liver cancer and breast cancer were the second and third most burdened cancer types attributed to high BMI in both China and globally in 2021, differing from those in 1990. Notably, liver cancer exhibited a concerning increase from 1990 to 2021, with a percentage change of 2.05 (1.13–3.07) in ASMR and 1.07 (0.78–1.32) in ASDR.

### 3.3 The influence of age, period, and cohort on the temporal trends of high BMI-attributed cancer burden in China and globally

**Figure 3:**
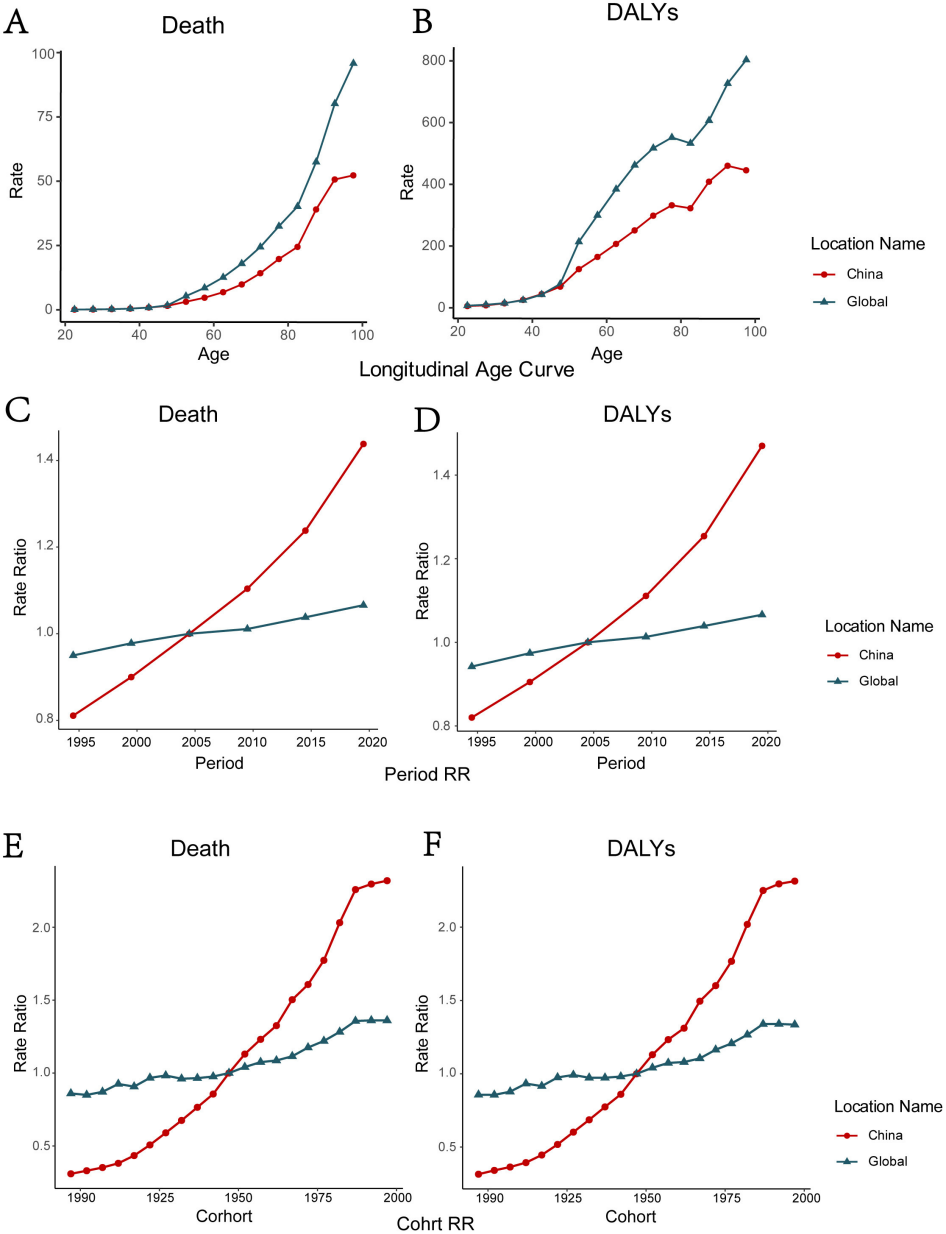
The age-period-cohort analysis of Death and DALYs due to high BMI-attributed cancer in China and global. The age effect of Death **(A)** and DALYs **(B)**; The period effect of Death **(C)** and DALYs **(D)**; The cohort effect of Death **(E)** and DALYs **(F)** in China **(E)**.

After adjusting for period and cohort effects, both the mortality and the DALYs rate of high BMI-attributed cancer increased with age in China and globally. After the age of 40, the rate of increase of age effect on high BMI-attributed cancer is particularly significant (Figures 3A, B; Supplementary Tables S3, S4). Throughout the observation period, the period effect on mortality and DALYs of high BMI-attributed cancer increased more rapidly in China compared with the global trend (Figures 3C, D; Supplementary Tables S5, S6). The cohort effect analysis revealed that the relative ratios of ASMR and ASDR increased more rapidly for individuals born before 1997 in China compared to the global trend (Figures 3E, F; Supplementary Tables S7, S8). Joinpoint regression analyses were employed to further explore the changes speed of ASMR and ASDR among younger (age < 40) and older (age ≥ 40) populations in different time segments (Supplementary Figure S3, Supplementary Tables S9–S12). Result showed that the ASDR and ASMR of young Chinese population are both higher than the global average. The APC of ASMR and ASDR among younger Chinese population is higher than that of the older population, which indicated a more rapid increasing burden in younger Chinese population.

### 3.4 Decomposition analysis on high BMI-attributed cancer mortality number and DALYs

**Figure 4:**
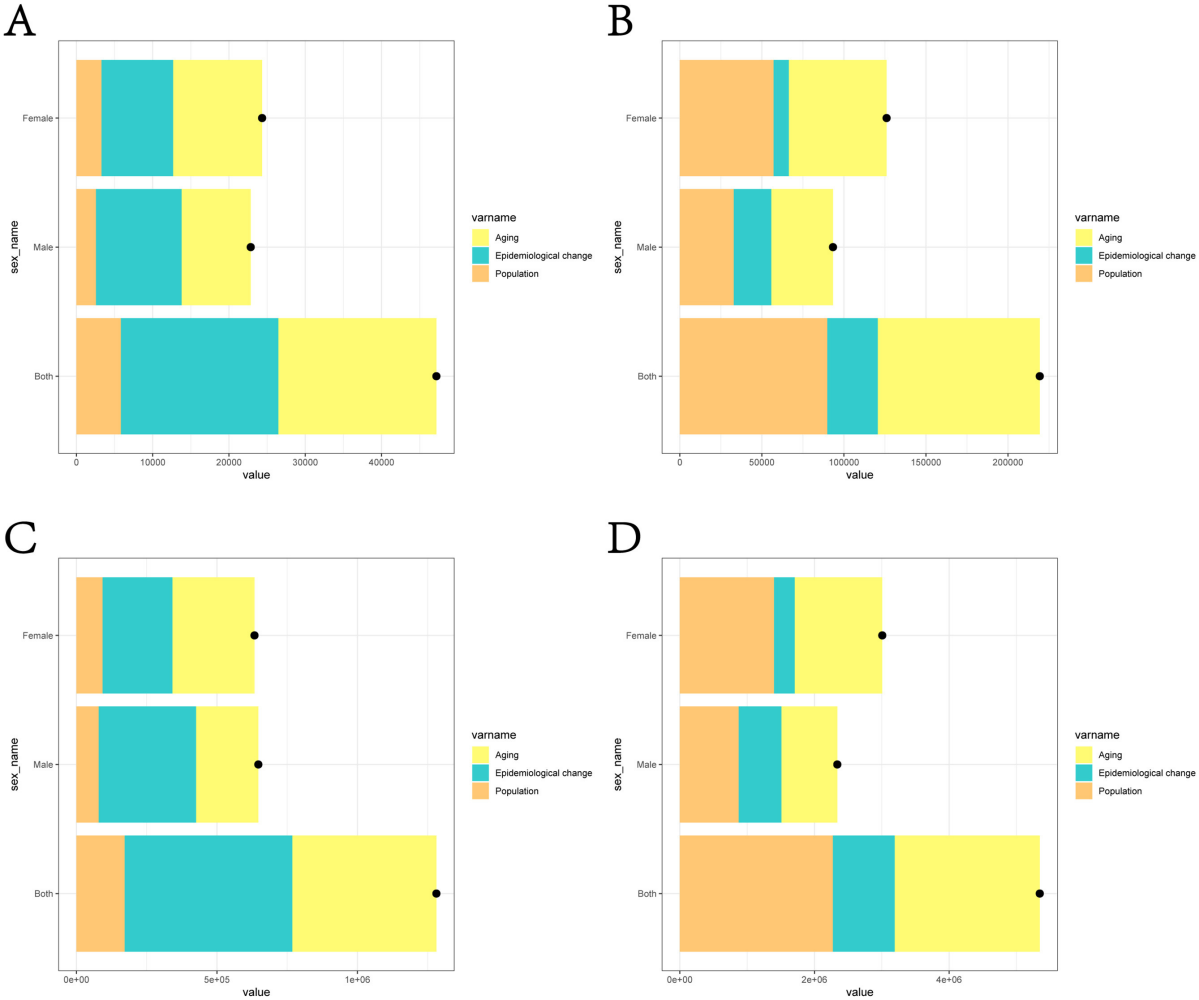
Analysis of high BMI-attributed cancer related deaths and DALYs from 1990 to 2021, considering the impact of population growth, aging, and epidemiological shifts in China and worldwide. **(A)** The changes of high BMI-attributed cancer's deaths cases in China; **(B)** The changes of high BMI-attributed cancer's DALYs in China; **(C)** The change of high BMI-attributed cancer's deaths cases in global; **(D)** The change of high BMI-attributed cancer's DALYs in global.

We performed decomposition analyses by sex, age structure, and epidemiological changes to thoroughly understand the factors influencing high BMI-attributed cancer burden shifts from 1990 to 2021 (Supplementary Tables S13, S14). As depicted in Figure 4, the decomposition analysis suggests that aging was a common and serious reason for the increased burden of high BMI-attributed cancer not only in China but the international community. In China, 43.92% of the increasing of high BMI-attributed cancer's deaths and 40.03% of the increasing of high BMI-attributed cancer's DALYs were due to aging. Globally, 44.99% of the increasing of high BMI-attributed cancer's deaths and 40.31% of the increasing of high BMI-attributed cancer's DALYs were due to aging. Epidemiological change played a significant role in the increasing burden of high BMI-attributed cancer in China. In China, 43.72% and 46.56% of the increase of high BMI-attributed cancer's deaths and high BMI-attributed cancer's DALYs were due to epidemiological change. From 1990 to 2021, epidemiological changes of high BMI-attributed cancer in China caused 20,632 deaths and 596,485 DALYs, accounting for 67.1% and 65.0% of the global total deaths and DALYs of high BMI-attributed cancer attributable to epidemiological changes. From a global perspective, the increase of deaths and DALYs of high BMI-attributed cancer caused by population growth accounted for 40.98% and 42.51%, respectively. While in China, only 13.4% and 12.36% of the increase of high BMI-attributed cancer's deaths and high BMI-attributed cancer's DALYs were due to population growth. Furthermore, the analysis by gender demonstrated the deaths and DALYs of high BMI-attributed cancer in male are 93,347 and 2,338,746 respectively. While the deaths and DALYs of high BMI-attributed cancer in female are 126,037 and 3,006,730, which were slightly higher than those in males. As for China, the deaths and DALYs of high BMI-attributed cancer were 22,848 and 647,327 in male, 24,340 and 633,648 in female. The difference by gender is not obvious in China.

### 3.5 Prediction of cancer burden attributed to high BMI in China and globally

**Figure 5:**
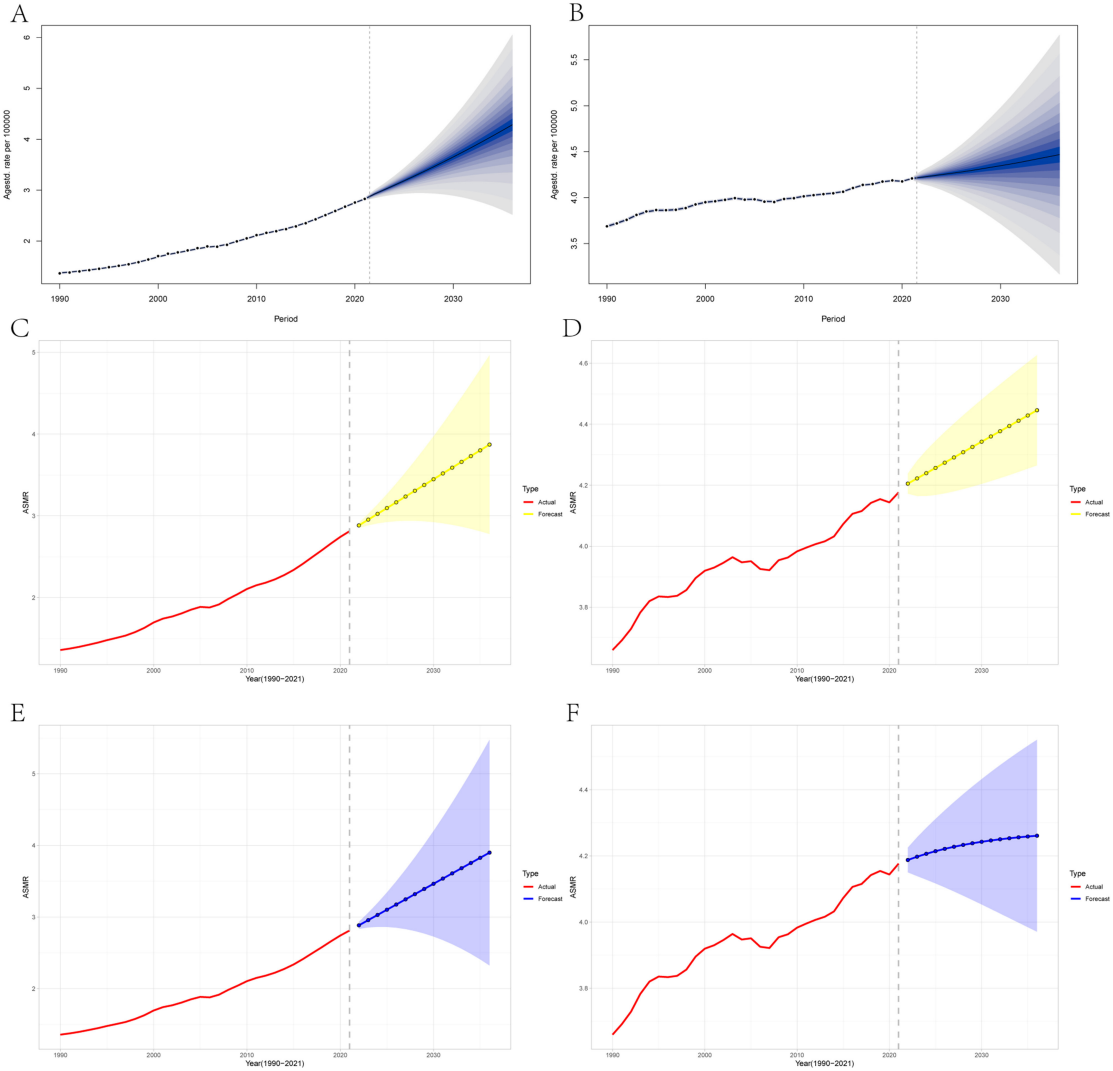
The temporal trends of high BMI-attributed cancer's ASMR in China and global between 1990 and 2036. **(A)** China, through BAPC model; **(B)** Global, through BAPC model; **(C)** China, through ARIMA model; **(D)** Global, through ARIMA model; **(E)** China, through ETS model; **(F)** Global, through ETS model.

Finally, we conducted BAPC, ARIMA, and ETS models to project the future trends of high BMI-attributed cancer from 2022 to 2036 in China and globally. The findings are detailed in Figure 5, Supplementary Figure S4, and Supplementary Tables S15–S17. All of the three models predict that ASMR and ASDR are projected to rise in China in the coming years (Figure 5; Supplementary Figure S4). The BAPC model indicated that the indexes (RMSE, MAE, and MAPE) in the BAPC model were better than those of the other models (Table 2). According to the BAPC model, China's ASMR and ASDR are expected to reach 4.29/105 and 124.39/105 respectively by 2036, while globally, these rates are expected to rise to 4.47/105 and 112.13/105 (Supplementary Table S15). Compared to China, the increase of ASMR and ASDR in globally are relatively modest.

**Table 2:** Performance evaluation of BAPC, ARIMA, and ETS model.

**Model**	**RMSE**	**MAE**	**MAPE**
BAPC	1.18156E-05	1.11537E-05	0.018935801
ARIMA	0.199759993	0.149386515	0.4250794
ETS	0.205366985	0.155851385	0.436700075

BAPC, Bayesian age-period-cohort; ARIMA, Auto-Regressive Moving Average Model; ETS, exponential smoothing model; RMSE, root mean square error; MAE, mean absolute error; MAPE, mean absolute percentage error.

## 4 Discussion

This study analyzes the trends in high BMI-attributed cancer burden in China over the past 30 years and projects them for the next 15 years from a global viewpoint. It offers a novel comparative analysis of GBD 2021 data on the burden of high BMI-attributed cancer in China and globally. The comparative analysis seeks to discern unique characteristics amid commonalities and explore methodological pathways through disparities.

Dai et al. (13) utilized GBD 2017 data to study the global impact of diseases attributed to elevated BMI. However, their attention to high BMI-attributed cancer was limited. Additionally, significant variations were observed in the data following the release of GBD 2021. The GBD study reported the ASMR and ASDR for high BMI-attributed cancer globally in 2017 as 5.8 (95% CI: 3.3, 8.9) and 133.4 (95% CI: 76.5, 205.7), respectively. In 2021, these figures were updated to 4.12 (95% CI: 1.67, 6.66) for ASMR and 100.24 (95% CI: 41.96, 160.68) for ASDR. This discrepancy may be attributed to enhancements in algorithms and models. To avoid skewed interpretations of high BMI-attributed cancer, it is essential to evaluate it using the latest data release. Additionally, recent findings by Liu et al. (36) derived from a population-based cancer registry in China confirmed our findings to some extent. The Chinese database is relatively comprehensive but limited in geographical coverage. To enable comparative analysis with international data, it is crucial to maintain consistent data collection and standardization methods, ensuring data comparability and consistency across various countries and regions. In this study, we used the latest GBD data to analyze the trends of high BMI-attributed cancer in China and globally from 1990 to 2021.

This study identified a rising trend in high BMI as a cancer risk factor over the past three decades. While China's ASMR and ASDR of high BMI-attributed cancer remain below the global average, their more pronounced growth rates reflect the alarming health impact of high BMI-attributed cancer. Significant international variations in the period effect of high BMI-attributed cancer suggest that high BMI is increasingly contributing to the cancer burden in China. The variation in the period effect of high BMI-attributed cancer may be attributed to differing risk exposures. The rapid urbanization in Asia has contributed to sedentary lifestyles and over-nutrition, fostering an obesity epidemic (37). The 2011 China Health and Nutrition Survey indicated a significant increase in obesity prevalence among Chinese adults in recent decades (38, 39). Chinese children could face significant issues with severe obesity. A national study in China found that 14.4% of children and adolescents are overweight (40, 41). Conversely, our study demonstrated that while the global burden of high BMI-attributed cancer remains relatively high, there has been a notable containment of its growth trend, suggesting that preventive measures for high BMI may be implemented in certain countries have yielded positive results. Previous studies have affirmed in countries like Germany (42), Italy (43), and Bahamas (44), comprehensive measures for prevention have successfully promoted the decline of BMI. These international strategies provide valuable insights for China's policymakers. By adapting and integrating these successful models, China can develop a multifaceted approach targeted high BMI and upstream factors to mitigate the growing burden of high BMI-attributed cancer. Additionally, the widespread adoption of medical examinations in China in recent years has led to the early detection of more cancer cases, partially explaining the significant rise in the burden of high BMI-attributed cancer.

Decomposition analysis identified aging as a major contributor to the rising cancer burden in both China and globally. The age-period-cohort model further revealed an increasing age effect in both areas, suggesting that aging may significantly influence the trend of high BMI-attributed cancer. These are particularly troubling results. Studies predict that by 2050, China's population of individuals aged 65 and older will reach 400 million, with 150 million being 80 years or older (45). Consequently, tackling the effects of an aging population on the public healthcare system presents a major challenge not only for China but globally. As a country with one of the largest aging populations in the world, China has already implemented a series of comprehensive measures to address the challenges posed by an aging society. For instance, the government has been actively promoting the development of community based older adult care services, integrating medical care and older adult care, and establishing a multi-level pension insurance system (46). Given the universality of aging related cancer burden, China's experience in implementing innovative policies and interventions may offer new strategies and perspectives for the international community. Future research could focus on evaluating the effectiveness of these policies. What's more, in our age stratified exploration, we found that the burden growth among Chinese young people is much faster compared to the global average. China's aging population has not yet reached its peak, and although the young population is relatively smaller, the ASDR and ASMR are increasing. This indicates that China will face this dual challenge of aging and disease youthification for a long time in the future. Therefore, it is particularly important to adopt age—specific measures tailored to the characteristics of different age groups.

Previous research has indicated that a higher BMI is a risk factor for cancer and contributes to cancer progression (47–50). For instance, high BMI boosts Interleukin-6 and tumor necrosis factor production, triggering hepatic inflammation and activating the oncogenic transcription factor STAT3 (signal transducer and activator of transcription 3), thereby driving liver cancer progression (51). What's more, high BMI is negatively correlated with high levels of CD8 cells, which was proved crucial in anti-tumor immunity (52). As a preventable risk factor, high BMI may become a key to the primary prevention of cancer. We present the latest high BMI-attributed cancer spectrum to inform and enhance public health strategies. In this latest high BMI-attributed cancer spectrum, colorectal cancer and liver cancer are found to be the first and second leading types of high BMI-attributed cancer. Therefore, greater focus should be placed on managing BMI, particularly for those with high risk of colorectal cancer and liver cancer, such as those with APC mutations, inflammatory bowel disease, and cirrhosis (11, 53–55).

However, several limitations exist in our study. Although GBD data are sourced from multiple origins and adjusted for accuracy, potential biases persist, necessitating validation via extensive epidemiological surveys. For example, GBD adopts disease burden models to deduce the lacking cancer data of low-income countries and this might result in excessively wide uncertainty intervals associated with deaths and DALYs. Therefore, while the point estimates offer valuable insights into trends and patterns, the wide confidence intervals underscore the need for caution when interpreting the magnitude and significance of these results. Secondly, the study did not consider certain potential covariates, such as smoking and alcohol consumption. Additionally, the GBD data were not categorized by urban-rural distinctions and tumor histology, restricting further analysis. At last, we explicitly acknowledge that the inability to fully isolate the impact of diagnostic practices improvements represents a significant limitation of the study.

## 5 Conclusion

Given the accelerated rise of high BMI-attributed cancer in China compared to global trends, there is an urgent need for effective, customized strategies for its primary prevention and management in the country. Future strategies should focus on creating preventive measures for individuals over 50 and those at risk of colorectal and liver cancer, particularly in relation to high BMI.

## Data Availability

The original contributions presented in the study are included in the article/Supplementary material, further inquiries can be directed to the corresponding authors.
