# Supplementary material for: Comparing the trends of cancer burden attributed to high BMI in China and globally from 1990 to 2021, with multi-model prediction to 2036
Source: Front Public Health. 2025 Jul 11;13:1590559. doi: 10.3389/fpubh.2025.1590559 (PMC12289581; doi:10.3389/fpubh.2025.1590559)
Supplement: Supplementary file 1 [file Data_Sheet_1.docx]

Supplementary Material

# Supplementary Figures


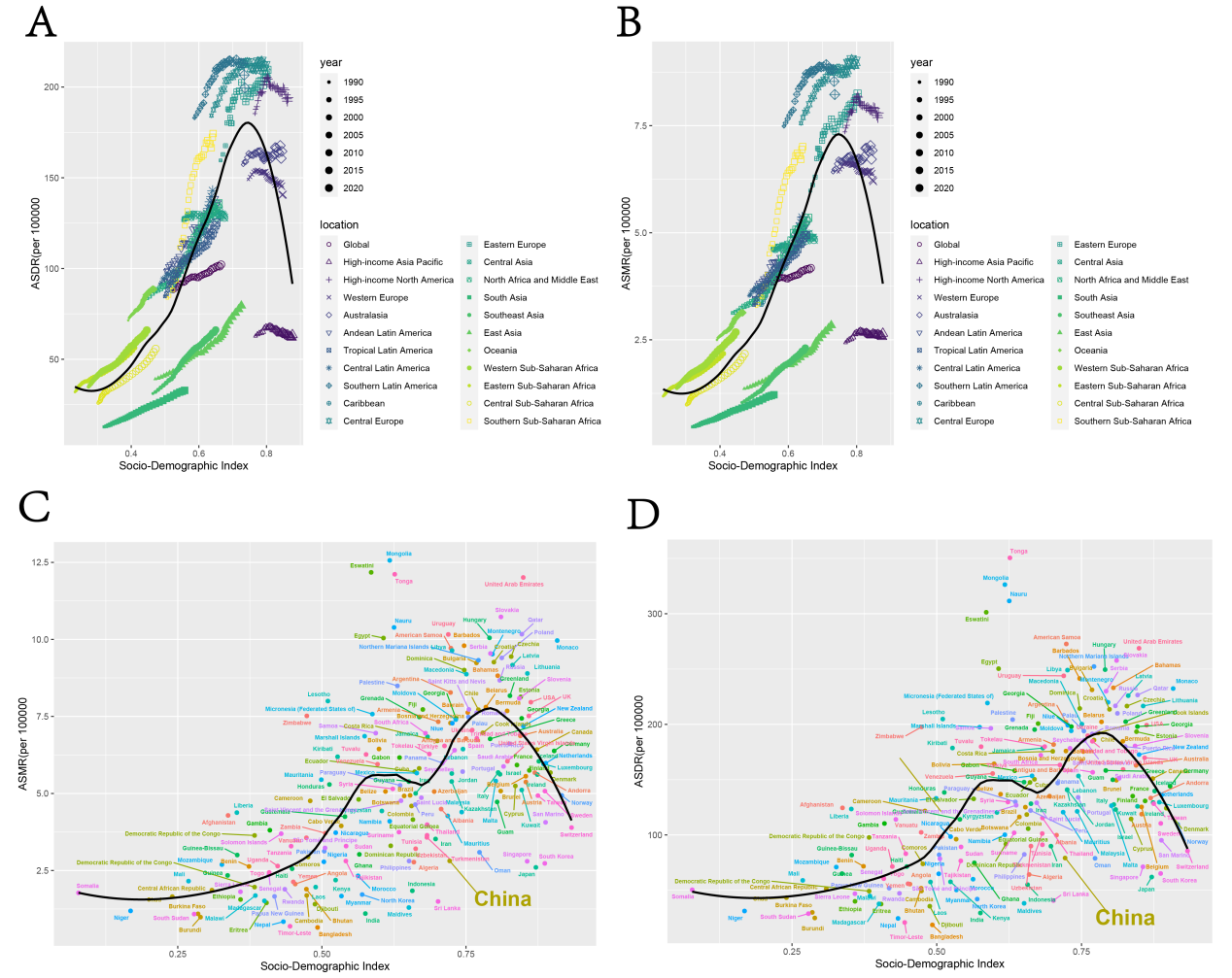


**Figure S1.** Trends in ASMR(A) and ASDR(B) of high body-mass index attributed cancer among different regions based on Socio-demographic index from 1990-2021; Trends in ASMR(C) and ASDR(D) due to HBAC of different countries by Socio-demographic index.ASMR,age-standardized mortality rate; ASDR, age-standardized DALYs rate.


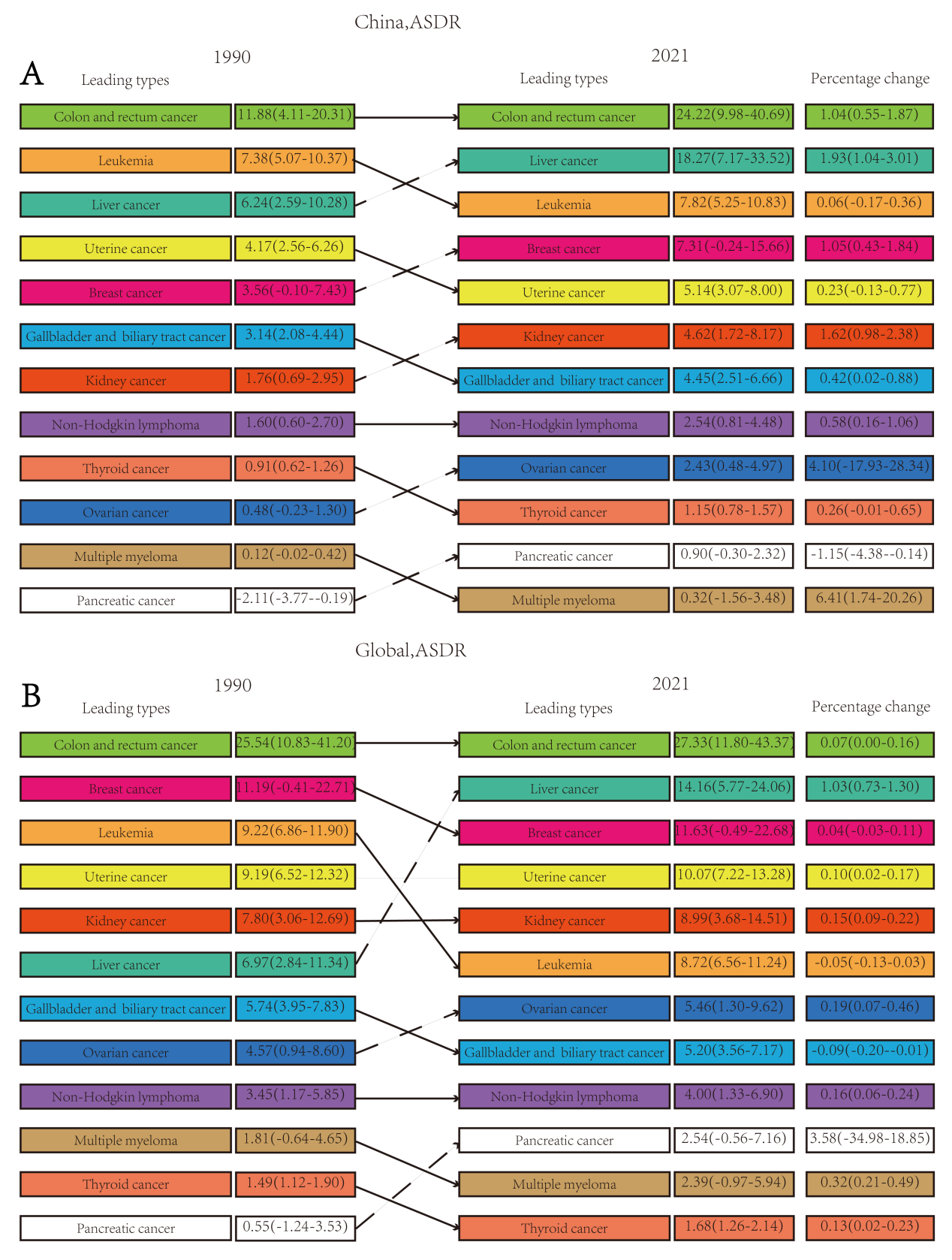


**Figure S2.** The leading types of cancer contribute to ASDR of high body-mass index attributed cancer in 1990 and 2021. The leading types of cancer contribute to ASDR of China(A) and world(B).ASMR,age-standardized mortality rate; ASDR, age-standardized DALYs rate;


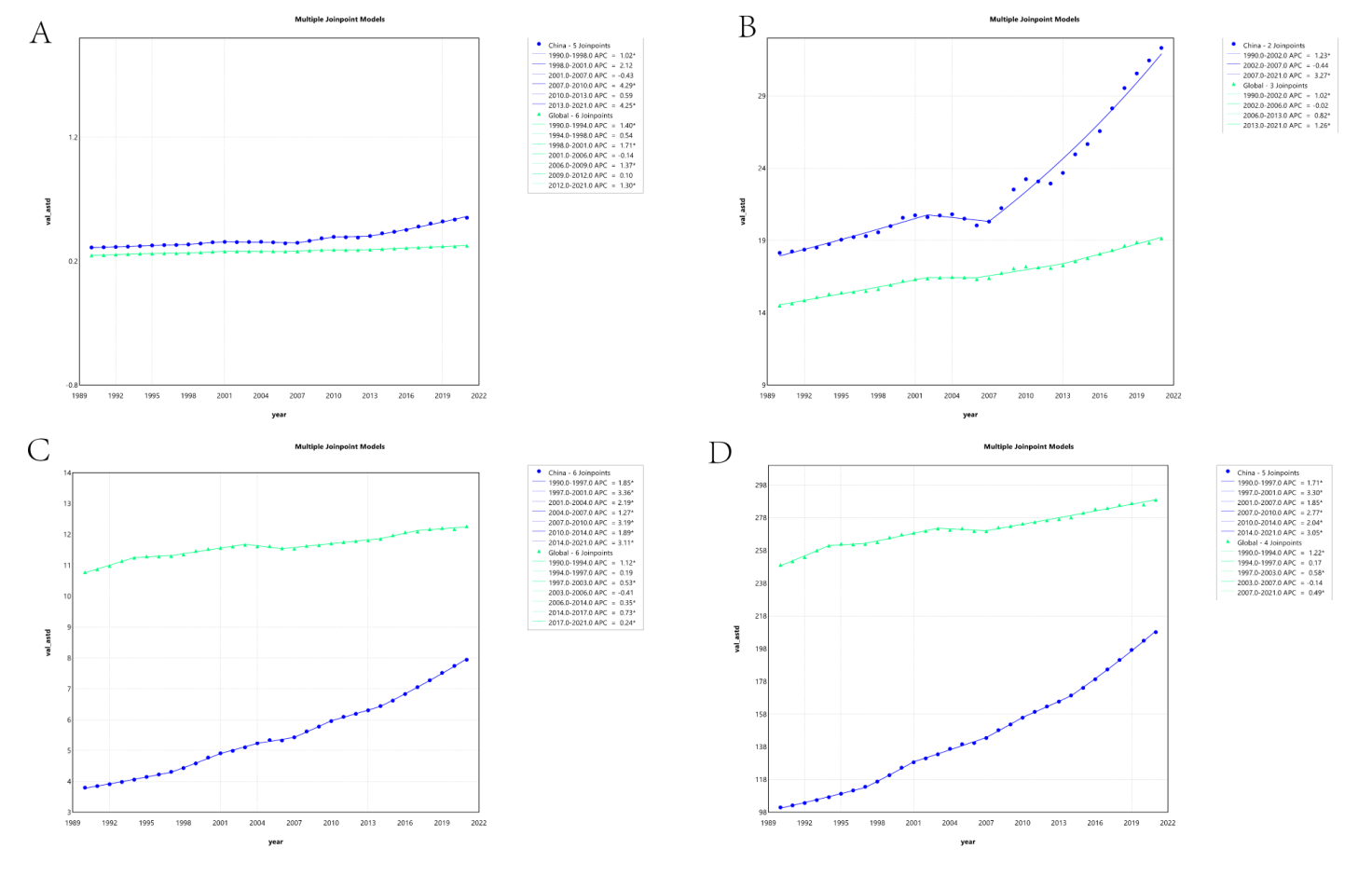


**Figure S3.** The APC of high BMI attributed cancer ASDR and ASMR among younger and older people in different segments from 1990 to 2021. (A) The APC of ASMR among people aged 20 to 39; (B) The APC of ASDR among people aged 20 to 39; (C) The APC of ASMR among people aged 40 and above; (D) The APC of ASDR among people aged 40 and above.


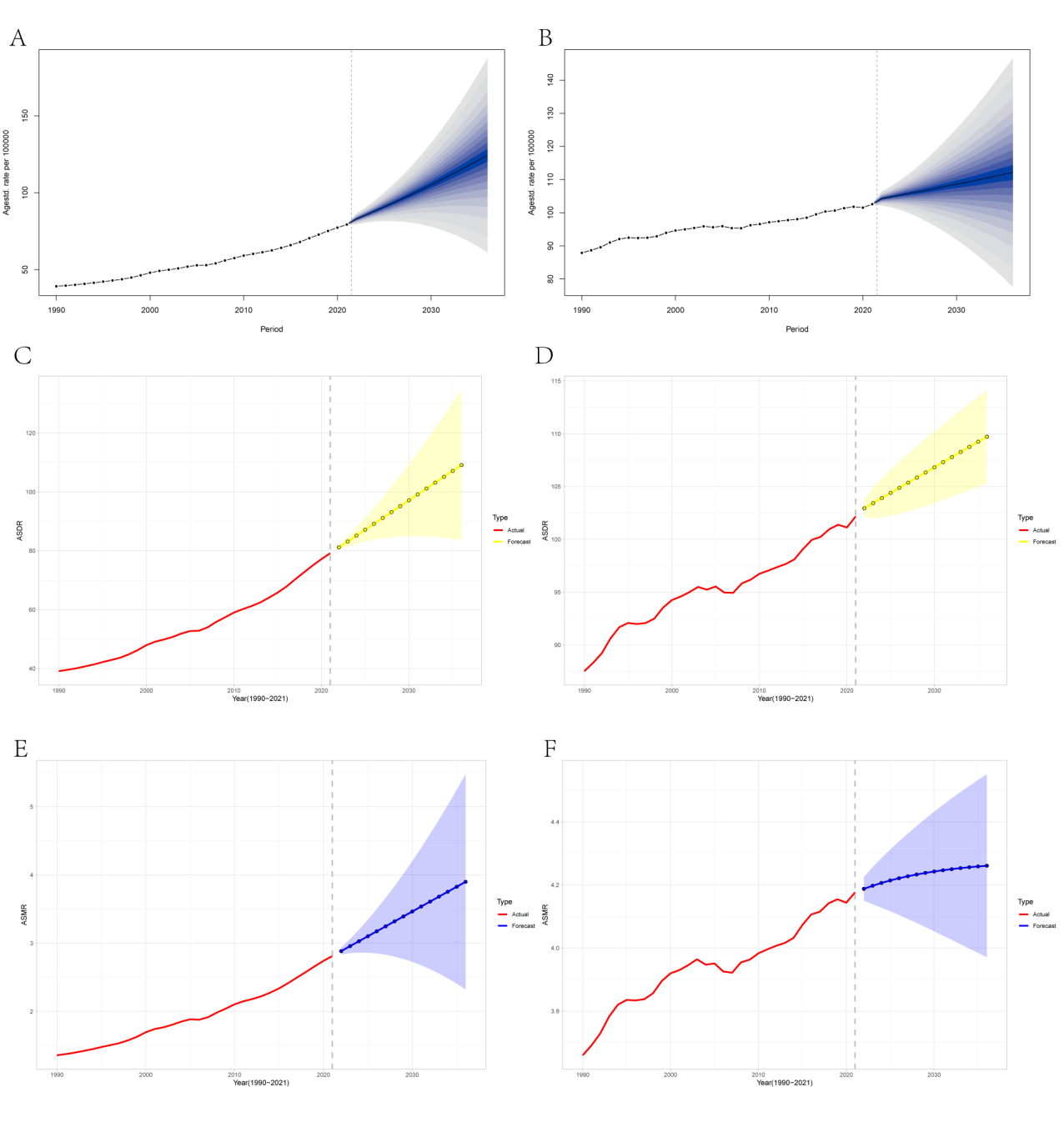


**Figure S4** The temporal trends of HBAC’s ASDR in China and global between 1990 and 2036. (A)China, through BAPC model; (B)global, through BAPC model; (C)China, through ARIMA model; (D)global, through ARIMA model; (E)China, through ETS model; (F)global, through ETS model.

# Supplementary Tables

**Table S1.** The APC of ASMR and ASDR for China of three segments from 1990-2021.

|  | ASMR | | |  | ASDR | | |
| --- | --- | --- | --- | --- | --- | --- | --- |
|  | 1990-1993 | 1993-2015 | 2015-2021 |  | 1990-1994 | 1994-2014 | 2014-2021 |
| APC | 1.57 | 2.3 | 3.17 |  | 1.48 | 2.2 | 3.14 |
| Lower CI | 0.01 | 2.21 | 2.57 |  | 0.68 | 2.12 | 2.75 |
| Upper CI | 3.16 | 2.38 | 3.77 |  | 2.28 | 2.28 | 3.54 |
| P-value | 0.049082 | < 0.000001 | < 0.000001 |  | 0.000788 | < 0.000001 | < 0.000001 |

## APC: annual percentage change; ASMR: age-standardized mortality rate; ASDR: age-standardized DALYs rate; Lower CI: lower confidence intervals; P-value: probability value.

**Table S2.** The APC of ASMR and ASDR for global of four segments form 1990-2021.

|  | ASMR | | | |  | ASDR | | | |
| --- | --- | --- | --- | --- | --- | --- | --- | --- | --- |
|  | 1990-1994 | 1994-2003 | 2003-2007 | 2007-2021 |  | 1990-1994 | 1994-2003 | 2003-2007 | 2017-2021 |
| APC | 1.02 | 0.43 | -0.23 | 0.46 |  | 1.09 | 0.48 | -0.08 | 0.51 |
| Lower CI | 0.81 | 0.35 | -0.5 | 0.42 |  | 0.86 | 0.39 | -0.36 | 0.47 |
| Upper CI | 1.38 | 0.52 | 0.01 | 0.5 |  | 1.51 | 0.58 | 0.18 | 0.57 |
| P-value | < 0.000001 | 0.0016 | 0.059588 | 0.0004 |  | < 0.000001 | 0.003999 | 0.563487 | 0.003199 |

APC: annual percentage change; ASMR: age-standardized mortality rate; ASDR: age-standardized DALYs rate; Lower CI: lower confidence intervals; P-value: probability value.

| **Table S3.** The age effect rate and it’s 95% confidence interval of high BMI related cancer deaths. | | | | | | | |
| --- | --- | --- | --- | --- | --- | --- | --- |
| Age | China | | |  | Global | | |
|  | Rate | CILo | CIHi |  | Rate | CILo | CIHi |
| 22.5 | 0.083 | 0.075 | 0.092 |  | 0.104 | 0.099 | 0.11 |
| 27.5 | 0.127 | 0.117 | 0.137 |  | 0.159 | 0.152 | 0.166 |
| 32.5 | 0.252 | 0.237 | 0.268 |  | 0.262 | 0.254 | 0.271 |
| 37.5 | 0.481 | 0.458 | 0.505 |  | 0.463 | 0.451 | 0.475 |
| 42.5 | 0.914 | 0.877 | 0.951 |  | 0.885 | 0.867 | 0.904 |
| 47.5 | 1.568 | 1.516 | 1.622 |  | 1.767 | 1.739 | 1.796 |
| 52.5 | 3.18 | 3.093 | 3.27 |  | 5.381 | 5.321 | 5.442 |
| 57.5 | 4.755 | 4.637 | 4.877 |  | 8.563 | 8.48 | 8.648 |
| 62.5 | 6.91 | 6.749 | 7.075 |  | 12.684 | 12.572 | 12.798 |
| 67.5 | 9.932 | 9.709 | 10.16 |  | 18.045 | 17.892 | 18.199 |
| 72.5 | 14.306 | 13.982 | 14.637 |  | 24.493 | 24.284 | 24.703 |
| 77.5 | 19.834 | 19.26 | 20.424 |  | 32.584 | 32.252 | 32.919 |
| 82.5 | 24.562 | 23.728 | 25.426 |  | 40.271 | 39.818 | 40.728 |
| 87.5 | 39.125 | 37.492 | 40.829 |  | 57.595 | 56.861 | 58.338 |
| 92.5 | 50.791 | 47.511 | 54.296 |  | 80.278 | 78.986 | 81.592 |
| 97.5 | 52.399 | 45.078 | 60.909 |  | 95.948 | 93.576 | 98.38 |
| CILo：Lower limit of the 95% confidence interval；CIHi：Higher limit of the 95% confidence interval | | | | | | | |

| **Table S4.** The age effect rate and it’s 95% confidence interval of high BMI related cancer DALYs. | | | | | | | |
| --- | --- | --- | --- | --- | --- | --- | --- |
| Age | China | | |  | Global | | |
|  | Rate | CILo | CIHi |  | Rate | CILo | CIHi |
| 22.5 | 5.681 | 5.243 | 6.155 |  | 7.045 | 6.776 | 7.324 |
| 27.5 | 8.045 | 7.52 | 8.606 |  | 10.029 | 9.708 | 10.361 |
| 32.5 | 14.748 | 13.965 | 15.575 |  | 15.305 | 14.894 | 15.727 |
| 37.5 | 25.809 | 24.652 | 27.02 |  | 24.787 | 24.235 | 25.352 |
| 42.5 | 44.65 | 42.936 | 46.433 |  | 43.134 | 42.345 | 43.937 |
| 47.5 | 68.92 | 66.617 | 71.303 |  | 77.673 | 76.527 | 78.836 |
| 52.5 | 125.686 | 122.052 | 129.429 |  | 214.206 | 211.848 | 216.59 |
| 57.5 | 165.371 | 160.856 | 170.012 |  | 300.254 | 297.253 | 303.286 |
| 62.5 | 207.596 | 202.053 | 213.292 |  | 384.934 | 381.262 | 388.641 |
| 67.5 | 251.499 | 244.672 | 258.517 |  | 462.631 | 458.197 | 467.108 |
| 72.5 | 299.203 | 290.42 | 308.25 |  | 518.021 | 512.726 | 523.371 |
| 77.5 | 332.998 | 319.963 | 346.563 |  | 552.62 | 545.572 | 559.76 |
| 82.5 | 323.393 | 306.885 | 340.789 |  | 533.807 | 525.6 | 542.143 |
| 87.5 | 409.548 | 380.558 | 440.746 |  | 607.624 | 595.833 | 619.649 |
| 92.5 | 461.188 | 404.33 | 526.042 |  | 727.647 | 707.533 | 748.333 |
| 97.5 | 446.574 | 323.8 | 615.9 |  | 804.304 | 766.555 | 843.911 |
| CILo：Lower limit of the 95% confidence interval；CIHi：Higher limit of the 95% confidence interval; DALYs: Disability-Adjusted Life Years. | | | | | | | |

| **Table S5.** The period effect rate and it’s 95% confidence interval of high BMI related cancer deaths. | | | | | | | |
| --- | --- | --- | --- | --- | --- | --- | --- |
| Period | China | | |  | Global | | |
|  | Rate Ratio | CILo | CIHi |  | Rate Ratio | CILo | CIHi |
| 1994.5 | 0.811 | 0.788 | 0.835 |  | 0.95 | 0.941 | 0.959 |
| 1999.5 | 0.9 | 0.877 | 0.923 |  | 0.978 | 0.97 | 0.986 |
| 2004.5 | 1 | 1 | 1 |  | 1 | 1 | 1 |
| 2009.5 | 1.104 | 1.079 | 1.129 |  | 1.011 | 1.003 | 1.019 |
| 2014.5 | 1.238 | 1.21 | 1.267 |  | 1.038 | 1.029 | 1.046 |
| 2019.5 | 1.438 | 1.403 | 1.474 |  | 1.066 | 1.508 | 1.075 |
| CILo：Lower limit of the 95% confidence interval；CIHi：Higher limit of the 95% confidence interval. | | | | | | | |

| **Table S6.** The period effect rate and it’s 95% confidence interval of high BMI related cancer DALYs. | | | | | | | |
| --- | --- | --- | --- | --- | --- | --- | --- |
| Period | China | | |  | Global | | |
|  | Rate Ratio | CILo | CIHi |  | Rate Ratio | CILo | CIHi |
| 1994.5 | 0.82 | 0.791 | 0.85 |  | 0.942 | 0.932 | 0.952 |
| 1999.5 | 0.905 | 0.878 | 0.932 |  | 0.974 | 0.965 | 0.984 |
| 2004.5 | 1 | 1 | 1 |  | 1 | 1 | 1 |
| 2009.5 | 1.111 | 1.081 | 1.141 |  | 1.013 | 1.004 | 1.023 |
| 2014.5 | 1.254 | 1.216 | 1.294 |  | 1.039 | 1.029 | 1.048 |
| 2019.5 | 1.47 | 1.415 | 1.526 |  | 1.066 | 1.056 | 1.076 |
| CILo：Lower limit of the 95% confidence interval；CIHi：Higher limit of the 95% confidence interval; DALYs: Disability-Adjusted Life Years. | | | | | | | |

| **Table S7.** The cohort effect rate and it’s 95% confidence interval of high BMI related cancer deaths. | | | | | | | |
| --- | --- | --- | --- | --- | --- | --- | --- |
| Cohort | China | | |  | Global | | |
|  | Rate Ratio | CILo | CIHi |  | Rate Ratio | CILo | CIHi |
| 1897 | 0.315 | 0.131 | 0.759 |  | 0.857 | 0.786 | 0.933 |
| 1902 | 0.341 | 0.259 | 0.447 |  | 0.856 | 0.823 | 0.89 |
| 1907 | 0.364 | 0.322 | 0.413 |  | 0.878 | 0.858 | 0.9 |
| 1912 | 0.394 | 0.364 | 0.426 |  | 0.933 | 0.917 | 0.949 |
| 1917 | 0.446 | 0.423 | 0.471 |  | 0.916 | 0.902 | 0.929 |
| 1922 | 0.518 | 0.497 | 0.539 |  | 0.975 | 0.963 | 0.987 |
| 1927 | 0.602 | 0.583 | 0.623 |  | 0.992 | 0.981 | 1.003 |
| 1932 | 0.686 | 0.667 | 0.707 |  | 0.973 | 0.963 | 0.983 |
| 1937 | 0.774 | 0.753 | 0.795 |  | 0.973 | 0.964 | 0.983 |
| 1942 | 0.86 | 0.838 | 0.882 |  | 0.981 | 0.972 | 0.99 |
| 1947 | 1 | 1 | 1 |  | 1 | 1 | 1 |
| 1952 | 1.13 | 1.102 | 1.158 |  | 1.041 | 1.031 | 1.051 |
| 1957 | 1.233 | 1.2 | 1.268 |  | 1.074 | 1.062 | 1.086 |
| 1962 | 1.311 | 1.27 | 1.353 |  | 1.08 | 1.066 | 1.095 |
| 1967 | 1.495 | 1.443 | 1.549 |  | 1.105 | 1.087 | 1.124 |
| 1972 | 1.601 | 1.53 | 1.674 |  | 1.164 | 1.137 | 1.192 |
| 1977 | 1.767 | 1.667 | 1.872 |  | 1.207 | 1.17 | 1.245 |
| 1982 | 2.019 | 1.875 | 2.173 |  | 1.266 | 1.216 | 1.317 |
| 1987 | 2.25 | 2.055 | 2.463 |  | 1.339 | 1.274 | 1.408 |
| 1992 | 2.295 | 2.007 | 2.625 |  | 1.34 | 1.252 | 1.435 |
| 1997 | 2.314 | 1.849 | 2.898 |  | 1.335 | 1.203 | 1.482 |
| CILo：Lower limit of the 95% confidence interval；CIHi：Higher limit of the 95% confidence interval. | | | | | | | |

| **Table S8.** The cohort effect rate and it’s 95% confidence interval of high BMI related cancer DALYs. | | | | | | | |
| --- | --- | --- | --- | --- | --- | --- | --- |
| Cohort | China | | |  | Global | | |
|  | Rate Ratio | CILo | CIHi |  | Rate Ratio | CILo | CIHi |
| 1897 | 0.309 | 0.046 | 2.058 |  | 0.86 | 0.724 | 1.022 |
| 1902 | 0.33 | 0.186 | 0.586 |  | 0.85 | 0.787 | 0.917 |
| 1907 | 0.352 | 0.275 | 0.452 |  | 0.871 | 0.834 | 0.908 |
| 1912 | 0.381 | 0.331 | 0.439 |  | 0.925 | 0.899 | 0.951 |
| 1917 | 0.434 | 0.398 | 0.474 |  | 0.907 | 0.888 | 0.927 |
| 1922 | 0.507 | 0.477 | 0.539 |  | 0.967 | 0.952 | 0.983 |
| 1927 | 0.59 | 0.564 | 0.619 |  | 0.983 | 0.97 | 0.996 |
| 1932 | 0.675 | 0.65 | 0.702 |  | 0.961 | 0.949 | 0.973 |
| 1937 | 0.765 | 0.739 | 0.791 |  | 0.965 | 0.955 | 0.976 |
| 1942 | 0.856 | 0.829 | 0.883 |  | 0.976 | 0.965 | 0.986 |
| 1947 | 1 | 1 | 1 |  | 1 | 1 | 1 |
| 1952 | 1.13 | 1.099 | 1.162 |  | 1.041 | 1.03 | 1.052 |
| 1957 | 1.231 | 1.194 | 1.269 |  | 1.075 | 1.062 | 1.087 |
| 1962 | 1.325 | 1.282 | 1.369 |  | 1.086 | 1.072 | 1.101 |
| 1967 | 1.503 | 1.45 | 1.558 |  | 1.115 | 1.098 | 1.133 |
| 1972 | 1.607 | 1.539 | 1.678 |  | 1.175 | 1.151 | 1.2 |
| 1977 | 1.773 | 1.681 | 1.869 |  | 1.22 | 1.188 | 1.252 |
| 1982 | 2.031 | 1.905 | 2.165 |  | 1.282 | 1.242 | 1.323 |
| 1987 | 2.258 | 2.094 | 2.436 |  | 1.357 | 1.306 | 1.41 |
| 1992 | 2.296 | 2.063 | 2.555 |  | 1.361 | 1.294 | 1.432 |
| 1997 | 2.319 | 1.952 | 2.756 |  | 1.361 | 1.262 | 1.467 |
| CILo：Lower limit of the 95% confidence interval；CIHi：Higher limit of the 95% confidence interval; DALYs: Disability-Adjusted Life Years. | | | | | | | |

| **Table S9.**  The APC of ASMR among people from age 20 to 39 in different segments from 1990 to 2021. | | | | | |
| --- | --- | --- | --- | --- | --- |
|  | | APC | Lower CI | Upper CI | P-value |
| China | 1990-1998 | 1.02 | 0.75 | 1.29 | <0.000001 |
|  | 1998-2001 | 2.12 | -0.37 | 4.67 | 0.090254 |
|  | 2001-2007 | -0.43 | -0.97 | 0.12 | 0.120721 |
|  | 2007-2010 | 4.29 | 1.64 | 7.02 | 0.003406 |
|  | 2010-2013 | 0.59 | -2.08 | 3.34 | 0.647573 |
|  | 2013-2021 | 4.253 | 3.92 | 4.59 | <0.000001 |
| Global | 1990-1994 | 1.4 | 1.03 | 1.76 | 0.000002 |
|  | 1994-1998 | 0.54 | -0.04 | 1.13 | 0.067205 |
|  | 1998-2001 | 1.72 | 0.5 | 2.92 | 0.009165 |
|  | 2001-2006 | -0.14 | -0.52 | 0.25 | 0.444543 |
|  | 2006-2009 | 1.37 | 0.11 | 2.65 | 0.035415 |
|  | 2009-2012 | 0.12 | -1.19 | 1.42 | 0.864005 |
|  | 2012-2021 | 1.3 | 1.18 | 1.43 | <0.000001 |
| APC: annual percentage change; ASMR: age-standardized mortality rate; ASDR: age-standardized DALYs rate; Lower CI: lower confidence intervals; P-value: probability value. | | | | | |

| **Table S10.** The APC of ASMR among people aged 40 and above in different segments from 1990 to 2021. | | | | | |
| --- | --- | --- | --- | --- | --- |
|  |  | APC | Lower CI | Upper CI | P-value |
| China | 1990-1997 | 1.85 | 1.74 | 1.96 | <0.000001 |
|  | 1997-2001 | 3.36 | 2.95 | 3.76 | <0.000001 |
|  | 2001-2004 | 2.19 | 1.39 | 3.00 | 0.000063 |
|  | 2004-2007 | 1.27 | 0.48 | 2.07 | 0.004334 |
|  | 2007-2010 | 3.19 | 2.36 | 4.02 | 0.000002 |
|  | 2010-2014 | 1.89 | 1.48 | 2.31 | <0.000001 |
|  | 2014-2021 | 3.11 | 2.99 | 3.23 | <0.000001 |
| Global | 1990-1994 | 1.12 | 0.90 | 1.34 | <0.000001 |
|  | 1994-1997 | 0.19 | -0.50 | 0.88 | 0.559855 |
|  | 1997-2003 | 0.53 | 0.38 | 0.69 | 0.000007 |
|  | 2003=2006 | -0.41 | -1.09 | 0.28 | 0.22151 |
|  | 2006-2014 | 0.35 | 0.26 | 0.44 | 0.000003 |
|  | 2014-2017 | 0.73 | 0.03 | 1.42 | 0.041123 |
|  | 2017-2021 | 0.24 | 0.02 | 0.46 | 0.036155 |
| APC: annual percentage change; ASMR: age-standardized mortality rate; ASDR: age-standardized DALYs rate; Lower CI: lower confidence intervals; P-value: probability value. | | | | | |

| **Table S11.**  The APC of ASDR among people from age 20 to 39 in different segments from 1990 to 2021. | | | | | |
| --- | --- | --- | --- | --- | --- |
|  |  | APC | Lower CI | Upper CI | P-value |
| China | 1990-2002 | 1.23 | 0.90 | 1.56 | <0.000001 |
|  | 2002-2007 | -0.44 | -2.13 | 1.28 | 0.602957 |
|  | 2007-2021 | 3.27 | 2.97 | 3.58 | <0.000001 |
| Global | 1990-2002 | 1.02 | 0.91 | 1.14 | <0.000001 |
|  | 2002-2006 | -0.02 | -1.04 | 1.02 | 0.971873 |
|  | 2006-2013 | 0.82 | 0.45 | 1.19 | 0.000134 |
|  | 2013-2021 | 1.26 | 1.00 | 1.51 | <0.000001 |
| APC: annual percentage change; ASMR: age-standardized mortality rate; ASDR: age-standardized DALYs rate; Lower CI: lower confidence intervals; P-value: probability value. | | | | | |

| **Table S12.** The APC of ASDR among people aged 40 and above in different segments from 1990 to 2021. | | | | | |
| --- | --- | --- | --- | --- | --- |
|  |  | APC | Lower CI | Upper CI | P-value |
| China | 1990-1992 | 1.28 | 0.19 | 2.37 | 0.024639 |
|  | 1992-1997 | 1.82 | 1.48 | 2.17 | <0.000001 |
|  | 1997-2001 | 3.25 | 2.69 | 3.81 | <0.000001 |
|  | 2001-2007 | 1.86 | 1.61 | 2.11 | <0.000001 |
|  | 2007-2010 | 2.77 | 1.64 | 3.91 | 0.000166 |
|  | 2010-2014 | 2.04 | 1.46 | 2.62 | 0.000005 |
|  | 2014-2021 | 3.05 | 2.88 | 3.21 | <0.000001 |
| Global | 1990-1994 | 1.22 | 0.94 | 1.49 | <0.000001 |
|  | 1994-1997 | 0.17 | -0.69 | 1.04 | 0.682816 |
|  | 1997-2003 | 0.58 | 0.39 | 0.78 | 0.000006 |
|  | 2003-2007 | -0.14 | -0.57 | 0.29 | 0.489377 |
|  | 2007-2021 | 0.49 | 0.44 | 0.53 | <0.000001 |
| APC: annual percentage change; ASMR: age-standardized mortality rate; ASDR: age-standardized DALYs rate; Lower CI: lower confidence intervals; P-value: probability value. | | | | | |

| **Table S13.** Decomposition analysis of Deaths and DALYs in China from 1990 to 2021. | | | | | | |
| --- | --- | --- | --- | --- | --- | --- |
|  | Deaths | | | DALYs | | |
|  | Both | Male | Female | Both | Male | Female |
| Overll diff | 47188.95 | 22848.7 | 24340.25 | 1280975.05 | 647327.01 | 633648.04 |
| a_effect | 20726.31 | 9058.52 | 11664.65 | 512795 | 221597.05 | 291975.42 |
| p_effect | 5830.32 | 2568.84 | 3289.1 | 171694.94 | 79321.61 | 92907.56 |
| r_effect | 20632.32 | 11221.34 | 9386.51 | 596485.1 | 346408.34 | 248765.06 |
| a_percent | 43.92 | 39.65 | 47.92 | 40.03 | 34.23 | 46.08 |
| p_percent | 12.36 | 11.24 | 13.51 | 13.4 | 12.25 | 14.66 |
| r_percent | 43.72 | 49.11 | 38.56 | 46.56 | 53.51 | 39.26 |
| val_1990 | 11556.25 | 4883.51 | 6672.74 | 377745.5 | 169432.20 | 208313.30 |
| val_2021 | 58745.20 | 27732.21 | 31013.00 | 1658720.6 | 816759.20 | 841961.30 |
| diff1 | 47188.95 | 22848.70 | 24340.25 | 1280975 | 647327.00 | 633648.00 |
| diff: difference; a_effect: aging factor effect; p_effect: population factor effect; r_effect: epidemiological factor effect; a_percent: aging factor percent; p_percent: population factor percent; r_percent: epidemiological factor percent; val: value | | | | | | |

| **Table S14.** Decomposition analysis of Deaths and DALYs in global from 1990 to 2021. | | | | | | |
| --- | --- | --- | --- | --- | --- | --- |
|  | Deaths | | | DALYs | | |
|  | Both | Male | Female | Both | Male | Female |
| Overll diff | 219385.35 | 93347.83 | 126037.51 | 5345475.53 | 2338745.71 | 3006729.82 |
| a_effect | 98705.46 | 37608.19 | 59585.19 | 2154855.9 | 829890.41 | 1301278.91 |
| p_effect | 89912.13 | 32875.78 | 57170.34 | 2272252.92 | 875161.84 | 1400664.96 |
| r_effect | 30767.76 | 22863.87 | 9281.99 | 918366.71 | 633693.45 | 304785.96 |
| a_percent | 44.99 | 40.29 | 47.28 | 40.31 | 35.48 | 43.28 |
| p_percent | 40.98 | 35.22 | 45.36 | 42.51 | 37.42 | 46.58 |
| r_percent | 14.02 | 24.49 | 7.36 | 17.18 | 27.10 | 10.14 |
| val_1990 | 137353.00 | 46511.69 | 90841.31 | 3549049.00 | 1290002.00 | 2259047.00 |
| val_2021 | 356738.30 | 139859.50 | 216878.80 | 8894525.00 | 3628748.00 | 5265777.00 |
| diff1 | 219385.35 | 93347.83 | 126037.51 | 5345476.00 | 2338746.00 | 3006730.00 |
| diff: difference; a_effect: aging factor effect; p_effect: population factor effect; r_effect: epidemiological factor effect; a_percent: aging factor percent; p_percent: population factor percent; r_percent: epidemiological factor percent; val: value | | | | | | |

| Table S15. The prediction and stantard deviation through BAPC of ASMR and ASDR for HBAC from 1990 to 2036. | | | | | | | | |
| --- | --- | --- | --- | --- | --- | --- | --- | --- |
|  | **China** | | | | **Global** | | | |
|  | **ASMR(per 100000)** | | **ASDR(per 100000)** | | **ASMR(per 100000)** | | **ASDR(per 100000)** | |
|  | **Mean** | **Standard Deviation** | **Mean** | **Standard Deviation** | **Mean** | **Standard Deviation** | **Mean** | **Standard Deviation** |
| 2022 | 2.92 | 0.04 | 82.98 | 1.69 | 4.22 | 0.04 | 104.27 | 1.05 |
| 2023 | 3.01 | 0.06 | 85.56 | 2.41 | 4.24 | 0.06 | 104.81 | 1.57 |
| 2024 | 3.10 | 0.09 | 88.20 | 3.39 | 4.25 | 0.08 | 105.35 | 2.25 |
| 2025 | 3.18 | 0.13 | 90.88 | 4.60 | 4.26 | 0.11 | 105.86 | 3.06 |
| 2026 | 3.27 | 0.17 | 93.61 | 6.02 | 4.28 | 0.15 | 106.36 | 3.96 |
| 2027 | 3.36 | 0.21 | 96.40 | 7.65 | 4.29 | 0.19 | 106.89 | 4.97 |
| 2028 | 3.46 | 0.26 | 99.26 | 9.47 | 4.31 | 0.23 | 107.48 | 6.06 |
| 2029 | 3.55 | 0.32 | 102.19 | 11.51 | 4.33 | 0.27 | 108.07 | 7.24 |
| 2030 | 3.65 | 0.39 | 105.17 | 13.75 | 4.35 | 0.32 | 108.63 | 8.49 |
| 2031 | 3.75 | 0.45 | 108.20 | 16.22 | 4.37 | 0.37 | 109.15 | 9.81 |
| 2032 | 3.85 | 0.53 | 111.30 | 18.90 | 4.38 | 0.42 | 109.71 | 11.21 |
| 2033 | 3.96 | 0.61 | 114.49 | 21.83 | 4.41 | 0.48 | 110.34 | 12.69 |
| 2034 | 4.07 | 0.70 | 117.75 | 25.00 | 4.43 | 0.54 | 110.99 | 14.25 |
| 2035 | 4.17 | 0.80 | 121.05 | 28.41 | 4.45 | 0.60 | 111.58 | 15.86 |
| 2036 | 4.29 | 0.90 | 124.39 | 32.07 | 4.47 | 0.66 | 112.13 | 17.54 |

ASMR: age-standardized mortality rate; ASDR: age-standardized DALYs rate.

| **Table S16**: The prediction and stantard deviation through ARIMA of ASMR and ASDR for HBAC from 1990 to 2036. | | | | | | | | | | | | |
| --- | --- | --- | --- | --- | --- | --- | --- | --- | --- | --- | --- | --- |
|  | **China** | | | | | | **Global** | | | | | |
|  | **ASMR(per 100000)** | | | **ASDR(per 100000)** | | | **ASMR(per 100000)** | | | **ASDR(per 100000)** | | |
|  | **Mean** | **Lower** | **Upper** | **Mean** | **Lower** | **Upper** | **Mean** | **Lower** | **Upper** | **Mean** | **Lower** | **Upper** |
| 2022 | 2.88 | 2.85 | 2.91 | 81.17 | 80.45 | 81.89 | 4.21 | 4.17 | 4.24 | 102.95 | 102.10 | 103.79 |
| 2023 | 2.95 | 2.88 | 3.02 | 83.16 | 81.55 | 84.78 | 4.22 | 4.16 | 4.28 | 103.43 | 102.00 | 104.86 |
| 2024 | 3.02 | 2.91 | 3.14 | 85.16 | 82.46 | 87.87 | 4.24 | 4.16 | 4.31 | 103.91 | 102.08 | 105.75 |
| 2025 | 3.09 | 2.92 | 3.26 | 87.16 | 83.20 | 91.12 | 4.26 | 4.17 | 4.35 | 104.40 | 102.23 | 106.57 |
| 2026 | 3.17 | 2.93 | 3.40 | 89.16 | 83.80 | 94.51 | 4.27 | 4.17 | 4.37 | 104.88 | 102.43 | 107.34 |
| 2027 | 3.24 | 2.94 | 3.53 | 91.15 | 84.26 | 98.05 | 4.29 | 4.18 | 4.40 | 105.37 | 102.65 | 108.08 |
| 2028 | 3.31 | 2.94 | 3.67 | 93.15 | 84.60 | 101.70 | 4.31 | 4.19 | 4.43 | 105.85 | 102.90 | 108.80 |
| 2029 | 3.38 | 2.93 | 3.82 | 95.15 | 84.83 | 105.47 | 4.33 | 4.20 | 4.46 | 106.34 | 103.17 | 109.50 |
| 2030 | 3.45 | 2.92 | 3.97 | 97.15 | 84.95 | 109.34 | 4.34 | 4.20 | 4.48 | 106.82 | 103.45 | 110.19 |
| 2031 | 3.52 | 2.91 | 4.13 | 99.14 | 84.97 | 113.32 | 4.36 | 4.21 | 4.51 | 107.31 | 103.74 | 110.87 |
| 2032 | 3.59 | 2.89 | 4.29 | 101.14 | 84.89 | 117.39 | 4.38 | 4.22 | 4.53 | 107.79 | 104.05 | 111.54 |
| 2033 | 3.66 | 2.87 | 4.45 | 103.14 | 84.72 | 121.56 | 4.39 | 4.23 | 4.55 | 108.28 | 104.36 | 112.19 |
| 2034 | 3.73 | 2.84 | 4.62 | 105.14 | 84.46 | 125.81 | 4.41 | 4.24 | 4.58 | 108.76 | 104.67 | 112.85 |
| 2035 | 3.80 | 2.81 | 4.79 | 107.13 | 84.12 | 130.15 | 4.43 | 4.25 | 4.60 | 109.24 | 105.00 | 113.49 |
| 2036 | 3.87 | 2.78 | 4.97 | 109.13 | 83.69 | 134.57 | 4.45 | 4.27 | 4.63 | 109.73 | 105.33 | 114.13 |

ASMR: age-standardized mortality rate; ASDR: age-standardized DALYs rate.

| **Table S17**: The prediction and stantard deviation through ETS of ASMR and ASDR for HBAC from 1990 to 2036. | | | | | | | | | | | | |
| --- | --- | --- | --- | --- | --- | --- | --- | --- | --- | --- | --- | --- |
|  | **China** | | | | | | **Global** | | | | | |
|  | **ASMR(per 100000)** | | | **ASDR(per 100000)** | | | **ASMR(per 100000)** | | | **ASDR(per 100000)** | | |
|  | **Mean** | **Lower** | **Upper** | **Mean** | **Lower** | **Upper** | **Mean** | **Lower** | **Upper** | **Mean** | **Lower** | **Upper** |
| 2022 | 2.88 | 2.84 | 2.93 | 81.17 | 80.04 | 82.29 | 4.19 | 4.15 | 4.22 | 102.63 | 101.72 | 103.53 |
| 2023 | 2.96 | 2.85 | 3.06 | 83.16 | 80.64 | 85.69 | 4.20 | 4.14 | 4.26 | 103.08 | 101.80 | 104.37 |
| 2024 | 3.03 | 2.86 | 3.20 | 85.16 | 80.91 | 89.42 | 4.21 | 4.13 | 4.28 | 103.54 | 101.97 | 105.11 |
| 2025 | 3.10 | 2.86 | 3.34 | 87.16 | 80.89 | 93.42 | 4.21 | 4.12 | 4.31 | 104.00 | 102.19 | 105.81 |
| 2026 | 3.17 | 2.85 | 3.50 | 89.16 | 80.62 | 97.69 | 4.22 | 4.11 | 4.34 | 104.46 | 102.43 | 106.48 |
| 2027 | 3.25 | 2.83 | 3.66 | 91.15 | 80.11 | 102.20 | 4.23 | 4.09 | 4.36 | 104.92 | 102.70 | 107.14 |
| 2028 | 3.32 | 2.81 | 3.83 | 93.15 | 79.37 | 106.93 | 4.23 | 4.08 | 4.39 | 105.37 | 102.98 | 107.77 |
| 2029 | 3.39 | 2.77 | 4.01 | 95.15 | 78.41 | 111.89 | 4.24 | 4.07 | 4.41 | 105.83 | 103.27 | 108.40 |
| 2030 | 3.46 | 2.73 | 4.20 | 97.15 | 77.24 | 117.05 | 4.24 | 4.05 | 4.43 | 106.29 | 103.57 | 109.01 |
| 2031 | 3.54 | 2.68 | 4.40 | 99.14 | 75.86 | 122.42 | 4.25 | 4.04 | 4.45 | 106.75 | 103.88 | 109.61 |
| 2032 | 3.61 | 2.62 | 4.60 | 101.14 | 74.29 | 128.00 | 4.25 | 4.03 | 4.47 | 107.21 | 104.20 | 110.21 |
| 2033 | 3.68 | 2.56 | 4.81 | 103.14 | 72.51 | 133.77 | 4.25 | 4.01 | 4.49 | 107.66 | 104.52 | 110.80 |
| 2034 | 3.75 | 2.49 | 5.02 | 105.14 | 70.54 | 139.73 | 4.26 | 4.00 | 4.51 | 108.12 | 104.85 | 111.39 |
| 2035 | 3.83 | 2.41 | 5.25 | 107.13 | 68.38 | 145.89 | 4.26 | 3.98 | 4.53 | 108.58 | 105.19 | 111.97 |
| 2036 | 3.90 | 2.32 | 5.47 | 109.13 | 66.03 | 152.23 | 4.26 | 3.97 | 4.55 | 109.04 | 105.53 | 112.55 |

ASMR: age-standardized mortality rate; ASDR: age-standardized DALYs rate.
